# A Quad-Constellation GNSS Navigation Algorithm with Colored Noise Mitigation

**DOI:** 10.3390/s19245563

**Published:** 2019-12-16

**Authors:** Xianqiang Cui, Tianhang Gao, Changsheng Cai

**Affiliations:** School of Geosciences and Info-Physics, Central South University, Changsha 410083, China; cuixianqiang@csu.edu.cn (X.C.); csugth@csu.edu.cn (T.G.)

**Keywords:** quad-constellation GNSS, colored noise, functional model fitting filter, single point positioning, navigation

## Abstract

The existence of colored noise in kinematic positioning will greatly degrade the accuracy of position solutions. This paper proposes a Kalman filter-based quad-constellation global navigation satellite system (GNSS) navigation algorithm with colored noise mitigation. In this algorithm, the observation colored noise and state colored noise models are established by utilizing their residuals in the past epochs, and then the colored noise is predicted using the models for mitigation in the current epoch in the integrated Global Positioning System (GPS)/GLObal NAvigation Satellite System (GLONASS)/BeiDou Navigation Satellite System (BDS)/Galileo navigation. Kinematic single point positioning (SPP) experiments under different satellite visibility conditions and road patterns are conducted to evaluate the effect of colored noise on the positioning accuracy for the quad-constellation combined navigation. Experiment results show that the colored noise model can fit the colored noise more effectively in the case of good satellite visibility. As a result, the positioning accuracy improvement is more significant after handling the colored noise. The three-dimensional positioning accuracy can be improved by 25.1%. Different satellite elevation cut-off angles of 10º, 20º and 30º are set to simulate different satellite visibility situations. Results indicate that the colored noise is decreased with the increment of the elevation cut-off angle. Consequently, the improvement of the SPP accuracy after handling the colored noise is gradually reduced from 27.3% to 16.6%. In the cases of straight and curved roads, the quad-constellation GNSS-SPP accuracy can be improved by 22.1% and 25.7% after taking the colored noise into account. The colored noise can be well-modeled and mitigated in both the straight and curved road conditions.

## 1. Introduction

The global navigation satellite system (GNSS) single point positioning (SPP) technology has been widely used in navigation since the advent of the Global Positioning System (GPS) [1,2]. For a long period of time, the SPP technology has been mainly implemented by a single constellation of GPS. With the revitalization of the GLObal NAvigation Satellite System (GLONASS), along with two newly emerging constellations, namely the BeiDou navigation satellite system (BDS) and the Galileo system, the combined navigation by joint use of GPS, GLONASS, BDS, and Galileo constellations has become a new trend [3,4,5]. 

The quad-constellation integrated positioning can make full use of the redundant observations to enhance the positioning accuracy and improve availability and reliability of position solutions due to the increased number of visible satellites [6,7]. But if the functional and stochastic models for the combined quad-constellation positioning cannot be accurately established, errors such as residual ionospheric, atmospheric, and multipath errors will severely affect the positioning solutions. These system errors can mostly be categorized as colored noise. Therefore, how to properly handle the colored noise is an important issue in the navigation.

The Kalman filter is a commonly used method in navigation data processing. In the classic Kalman filter, it is assumed that both the observation noise and state noise belong to the Gaussian white noise. But in the navigation, most observation noise and state noise belong to colored noise due to the complex observation environment [8]. Differing from the white noise, the colored noise has an uneven power spectral density function [8]. The existence of the colored noise will greatly degrade the accuracy and reliability of position solutions in the Kalman filter parameter estimation [9,10]. 

So far there have been a few methods developed to handle the colored noise. Generally, they can be divided into two categories: functional model compensation filters and stochastic model compensation filters. The functional model compensation filters include the state vector augmented filter [11,12] and functional model fitting filter [13,14,15]. The stochastic model compensation filters include the adaptive filter based on Sage windowing weights and variance component [16], and the adaptive robust filter based on classified adaptive factor adjustment [17]. Among these methods, the most straightforward way to deal with the colored noise is the state vector augmented filter [11,12], which models the colored noise as a constant or follows a variation rule at a certain time interval. In this method, the colored noise is estimated along with the other state parameters in the parameter estimation process. As a result, the colored noise is absorbed by the state parameter and its effect is mitigated. However, this method cannot be applied to handle the observation colored noise [12]. The functional model-fitting filter method establishes respective function models to fit the observation colored noise and state colored noise, and then to forecast and mitigate these colored noises [13,14,15]. Generally, in the Kalman filter processing, the observation colored noise and the state colored noise will mostly remain in their residuals. Thus, the observation residual sequence and state residual sequence can be used to model these colored noises [16,17]. The adaptive filter based on the Sage window weights and variance component directly estimates the covariance matrix of the observation colored noise and the state colored noise by employing observation residuals and state residuals as sample values of colored noise [16]. The method involving an adaptive robust filter based on classified adaptive factor adjustment treats the observation colored noise as abnormal errors and state colored noise as dynamic disturbance. By adjusting the observation weight to restrain the abnormal errors and using the adaptive factor to suppress the dynamic disturbance, the effect of the colored noise is mitigated [17,18,19,20]. In addition to the above stated methods that are based on linear models, some nonlinear model filter methods have also been proposed to deal with the colored noise [21,22]. Existing studies have demonstrated that all these methods can reduce the effect of the colored noise effectively. Among them, the functional model fitting filter can be simply and efficiently implemented. Additionally, the observation colored noise and the state colored noise can be extracted for rational analysis. 

In this study, GPS, GLONASS, BDS, and Galileo are jointly used for positioning solutions in navigation. Based on the observation residuals and the state prediction residuals, a quad-constellation SPP algorithm with colored noise mitigation is proposed. In this algorithm, the models of the observation colored noise and state colored noise are established by applying a functional model fitting filter method, and then the colored noises are compensated before the parameter estimation.

The remaining part of the paper is organized as follows. Section 2 describes the quad-constellation GNSS-SPP algorithm with colored noise mitigation. In Section 3, kinematic positioning experiments under different satellite visibility and different trajectory conditions are conducted to evaluate the performance improvement of the quad-constellation SPP algorithm. Finally, conclusions are drawn in Section 4.

## 2. Quad-Constellation GNSS-SPP Algorithm with Colored Noise Mitigation

The Kalman filter method is generally used for parameter estimation in the SPP. Since the quad-constellation GNSSs use different time scales, it is necessary to estimate each satellite system’s receiver clock offset with respect to their respective time scale, even if there is only one physical clock used in the multi-GNSS receiver. Instead of estimating receiver clock offset parameters by referring to their respective system time, the system time difference parameters with respect to a reference time scale can be introduced. If the GPS time scale is selected as this reference, the GPS receiver clock offset is directly estimated as an unknown parameter, while the receiver clock offset parameters for the other satellite systems can be depicted as the sum of the GPS receiver clock offset and the system time difference parameter. The quad-constellation GNSS-SPP observation model can be written as [6]:

(1) $$\left\{\begin{array}{l} P^{G}=\rho^{G}+c\cdot \delta t-c\cdot \delta t_{s}^{G}+I_{i}^{G}+T_{i}^{G}+d_{orb}^{G}+\varepsilon_{P}^{G}\\ P^{R}=\rho^{R}+c\cdot \delta t+c\cdot \delta t_{sys}^{R,G}-c\cdot \delta t_{s}{}^{R}+I_{i}^{R}+T_{i}^{R}+d_{orb}^{R}+\varepsilon_{P}^{R}\\ P^{E}=\rho^{E}+c\cdot \delta t+c\cdot \delta t_{sys}^{E,G}-c\cdot \delta t_{s}{}^{E}+I_{i}^{E}+T_{i}^{E}+d_{orb}^{E}+\varepsilon_{P}^{E}\\ P^{C}=\rho^{C}+c\cdot \delta t+c\cdot \delta t_{sys}^{C,G}-c\cdot \delta t_{s}{}^{C}+I_{i}^{C}+T_{i}^{C}+d_{orb}^{C}+\varepsilon_{P}^{C} \end{array}\right.$$

where the superscripts *G*, *R*, *E*, and *C* represent GPS, GLONASS, Galileo, and BDS, respectively; P is the measured pseudorange in meters; $\rho^{}$ is the geometric range in meters; c is the speed of light, δt is the GPS receiver clock offset in seconds; $\delta t_{s}^{}$ is the satellite clock offset in seconds; $\delta t_{sys}^{R,G}$, $\delta t_{sys}^{E,G}$, and $\delta t_{sys}^{C,G}$ are the GPS-GLONASS, GPS-Galileo, and GPS-BDS system time differences in seconds, respectively. Here, $I_{i}^{}$ is the ionospheric delay error in meters, $T_{i}^{}$ is the tropospheric delay error in meters, $d_{orb}^{}$ is the satellite orbit error in meters, $\varepsilon_{P}^{}$ is the measurement noise including multipath in meters. The hardware delay on the receiver end will be absorbed by the receiver clock offset and the system time difference parameters, whereas the hardware delay bias on the satellite end can be corrected by the group delay provided in the broadcast ephemeris. Thus, the hardware delay biases do not show up in Equation (1).

As an efficient realization of the sequential least-squares adjustment, the Kalman filter has been widely used in the GNSS navigation computations. In a discrete Kalman filter, the measurement equation and state equation may be written as:

(2) $$L_{k}=H_{k}x_{k}+e_{k}$$

(3) $$x_{k}=\Phi_{k,k-1}x_{k-1}+w_{k}$$

where $L_{k}$ is the observation vector; *k* is the epoch; $H_{k}$ is the design matrix; $e_{k}$ and $w_{k}$ are observation noise and state noise, respectively. Here, $x_{k}$ is the state vector to be estimated, including the position coordinates, velocity, receiver clock difference, and system time difference parameters; $\Phi_{k,k-1}$ is the state transition matrix; Δt is the time interval. The state vector and state transition matrix are represented as follows:

(4) $$x_{k}=[X\;Y\;Z\;V_{X}\;V_{Y}\;V_{Z}\;c\cdot \delta t\;c\cdot \delta t_{sys}^{R,G}\;c\cdot \delta t_{sys}^{E,G}\;c\cdot \delta t_{sys}^{C,G}{]}^{T}$$

(5) $$\Phi_{k,k-1}=\left[\begin{array}{cccc} 1 & 0 & 0 & \begin{array}{cccc} \Delta t & 0 & 0 & \begin{array}{cccc} 0 & 0 & 0 & 0 \end{array} \end{array}\\ 0 & 1 & 0 & \begin{array}{cccc} 0 & \Delta t & 0 & \begin{array}{cccc} 0 & 0 & 0 & 0 \end{array} \end{array}\\ 0 & 0 & 1 & \begin{array}{cccc} 0 & 0 & \Delta t & \begin{array}{cccc} 0 & 0 & 0 & 0 \end{array} \end{array}\\ \begin{array}{c} 0\\ 0\\ 0\\ \begin{array}{c} 0\\ 0\\ 0\\ 0 \end{array} \end{array} & \begin{array}{c} 0\\ 0\\ 0\\ \begin{array}{c} 0\\ 0\\ 0\\ 0 \end{array} \end{array} & \begin{array}{c} 0\\ 0\\ 0\\ \begin{array}{c} 0\\ 0\\ 0\\ 0 \end{array} \end{array} & \begin{array}{c} \begin{array}{cccc} 1 & 0 & 0 & \begin{array}{cccc} 0 & 0 & 0 & 0 \end{array} \end{array}\\ \begin{array}{cccc} 0 & 1 & 0 & \begin{array}{cccc} 0 & 0 & 0 & 0 \end{array} \end{array}\\ \begin{array}{cccc} 0 & 0 & 1 & \begin{array}{cccc} 0 & 0 & 0 & 0 \end{array} \end{array}\\ \begin{array}{c} \begin{array}{cccc} 0 & 0 & 0 & \begin{array}{cccc} 1 & 0 & 0 & 0 \end{array} \end{array}\\ \begin{array}{cccc} 0 & 0 & 0 & \begin{array}{cccc} 0 & 1 & 0 & 0 \end{array} \end{array}\\ \begin{array}{cccc} 0 & 0 & 0 & \begin{array}{cccc} 0 & 0 & 1 & 0 \end{array} \end{array}\\ \begin{array}{cccc} 0 & 0 & 0 & \begin{array}{cccc} 0 & 0 & 0 & 1 \end{array} \end{array} \end{array} \end{array} \end{array}\right]$$

If the $e_{k}$ and $w_{k}$ are not zero-mean Gaussian white noise, they can be considered as colored noises. A functional fitting model is adopted here to handle the colored noise. The functional models of the observation colored noise and state colored noise can be expressed as first-order autocorrelation models below [10]:

(6) $$e_{k}=\Psi_{k,k-1}\;e_{k-1}+\eta_{k}$$

(7) $$w_{k}=T_{k,k-1}w_{k-1}+\xi_{k}$$

where $\Psi_{k,k-1}$ and ${Τ}_{k,k-1}$ are diagonal coefficient matrices of the observation colored noise and state colored noise, respectively, $\eta_{k}$ and $\xi_{k}$ are zero-mean Gaussian white noise sequences. 

In the Kalman filter processing, the observation colored noise and the state colored noise mostly remain in the observation residuals and state residuals, respectively [16,17]. Thus, the observation residual sequence and state residual sequence can be used to predict the colored noise for correction in the next epoch. The fitting process of the observation colored noise is illustrated as an example in the following part.

Generally, the colored noise is correlated at consecutive epochs. Thus, the observation residuals of the previous *N* epoch can be used as sample data of the observation colored noise for function model fitting. For convenience of calculation, Equation (6) is transposed to:

(8) $$e_{k}^{T}=e_{k-1}^{T}\Psi_{k,k-1}+\eta_{k}^{T}$$

Then, a moving window technique is applied to update the sample data. Suppose $V_{k-N}\ldots V_{k-1}$ are *N* observation residuals before the current *k^th^* (*k > N*) epoch. In the navigation, the moving window size *N* will affect the fitting effect of the colored noise. If the window size is too large, the error correlation between the preceding epochs and the current epoch becomes weak, and thus the derived colored noise is not accurate. If the window size is too small, it could be too random to model the colored noise. Empirically, the window size can be set to 4–12. If these observation residuals are substituted into Equation (6), the error equation can be written as follows:

(9) $$r=B{\hat{\Psi }}_{k,k-1}{}^{-l}$$

where, $r=\left[\begin{array}{c} r_{k-1}\\ r_{k-2}\\ \vdots \\ r_{k-N+1} \end{array}\right],B=\left[\begin{array}{c} V_{k-2}^{T}\\ V_{k-3}^{T}\\ \vdots \\ V_{k-N}^{T} \end{array}\right],l=\left[\begin{array}{c} V_{k-1}^{T}\\ V_{k-3}^{T}\\ \vdots \\ V_{k-N+1}^{T} \end{array}\right]$, *r* is the error matrix of the fitted colored noise sequence, *l* is the observation matrix for the observation colored noise, *B* is the matrix of colored noise sequences at the epochs from *k−2* to *k−N*, and ${\hat{\Psi }}_{k,k-1}$ is the correlation coefficient matrix of the observation colored noise model. The sign above the $\Psi_{k,k-1}$ denotes estimated value.

According to the least-squares criterion, the error matrix *r* in Equation (9) should satisfy the minimization condition in Equation (10), and the coefficient matrix ${\hat{\Psi }}_{k,k-1}$ can be obtained as Equation (11):

(10) $$E\{{\left(r-E(r)\right)}^{T}(r-E(r))\}=\min$$

(11) $${\hat{\Psi }}_{k,k-1}={\left(B^{T}B\right)}^{-1}B^{T}l$$

where *E* ( ) is the statistical expectation. After the coefficient matrix of the observation colored noise is obtained, the observation colored noise estimate can be predicted using Equation (8) by replacing the observation colored noise $e_{k-1}$ with the observation residual $V_{k-1}$.

(12) $${\hat{e}}_{k}={\left(B^{T}B\right)}^{-1}B^{T}lV_{k-1}$$

Similarly, the state colored noise ${\hat{w}}_{k}$ can also be predicted. After obtaining the colored noise, the observation value and prediction state value are modified by applying the correction of the colored noise accordingly. The observation equation and state equation with colored noise correction are expressed as follows:

(13) $$L_{k}+{\hat{e}}_{k}=H_{k}x_{k}+\eta_{k}$$

(14) $$x_{k}=(\Phi_{k,k-1}x_{k-1}+{\hat{w}}_{k})+\xi_{k}$$

**Figure 1:**
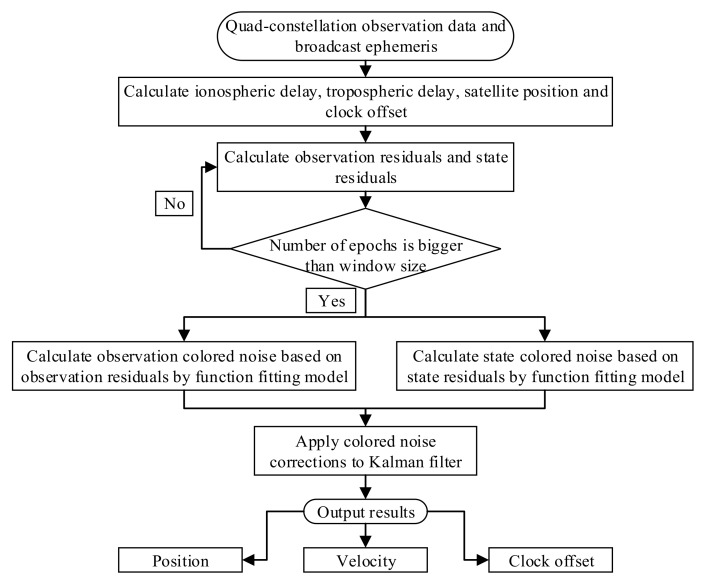
Quad-constellation global navigation satellite system single point positioning (GNSS-SPP) algorithm with mitigation of colored noise.

The Kalman filter solution can be obtained using Equations (13) and (14). The flow chart of the quad-constellation SPP solutions with mitigation of colored noise is shown in Figure 1. First, the quad-constellation observation data and broadcast ephemeris are collected in the navigation. Then, the ionospheric delay and tropospheric delay are corrected using the Klobuchar ionospheric model and Saastamoinen tropospheric model, respectively [23,24]. The satellite position and satellite clock offset are calculated using broadcast ephemerides. Next, the standard Kalman filter method is used to get observation and state residuals. Subsequently, the observation colored noise and state colored noise are predicted by a moving-window functional model, as shown in Equations (9)–(12). Finally, the colored noise is corrected in the Kalman filter, as shown in Equations (13) and (14), and the Kalman filter position solution is obtained.

## 3. Navigation Test Results and Analysis 

**Figure 2:**
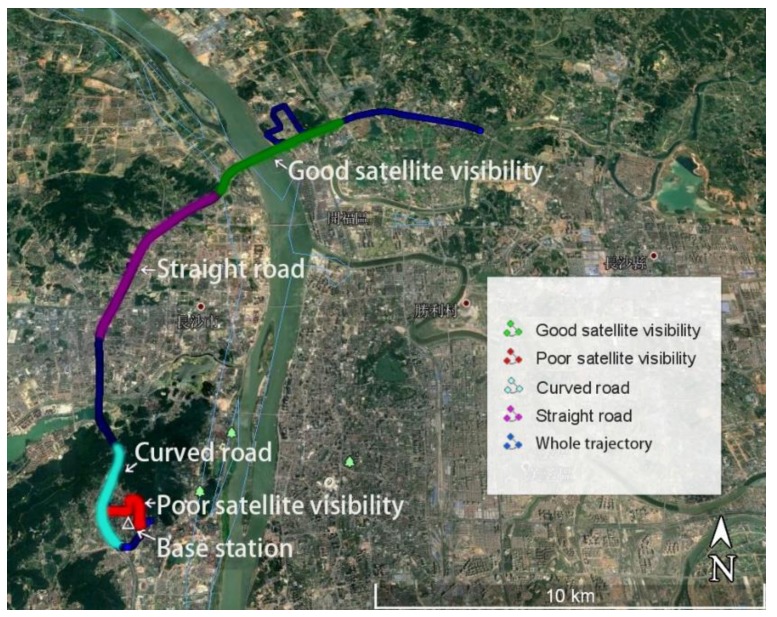
Vehicle running route produced by Google Earth for the quad-constellation navigation test.

**Figure 3:**
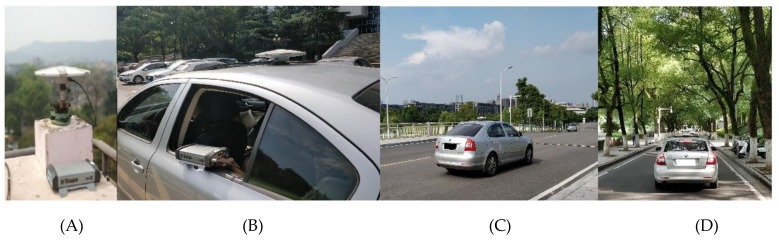
Equipment setup and navigation test environment: (**A**) Base station; (**B**) Rover station; (**C**) Open-sky road; (**D**) Signal blocked road.

To test the quad-constellation GNSS-SPP algorithm with mitigation of colored noise, a kinematic navigation experiment was conducted on March 28, 2018, in Changsha. The test started at the local time of 09:30:45 (GPS time of 01:30:45) and lasted for about 2 h, using a land vehicle that carried a Trimble Net R9 GNSS dual-frequency receiver, which can simultaneously collect datasets from quad-constellations of GPS, GLONASS, BDS, and Galileo with a sampling interval of 1 s. The receiver antenna type is a TRM55971.00, which is connected to the receiver via the sunroof and placed on the car roof. The position reference values of the car route are calculated using the real-time kinematic (RTK) technique [25]. RTKlib 2.4.3 software is used in a post-processing mode to obtain the position solutions at a three-dimensional (3D) accuracy of a few centimeters. The base station for the RTK processing is installed on the roof of the Wenfa building at Central South University. The receiver and antenna at the base station are the same as the rover station. The distance between the base station and the rover station is shorter than 16 kilometers. The sampling interval is set to 1s. The entire test route is shown in Figure 2, which is produced by Google Earth. The equipment setup and road visibility are shown in Figure 3. After staying for a period of time in front of the library on the main campus of Central South University, the car started to move on the campus slowly. After 10 minutes, the car ran into a fast lane (i.e. the South 2nd Ring road in Changsha). The Central South University campus is heavily wooded, which causes serious signal blocking. By contrast, there are almost no obstructions on the South 2nd Ring road, whereas there exists signal loss when the car passes some overpasses. In order to test the positioning performance in different road and visibility conditions, four different datasets are collected, including datasets collected on the straight road, on the curved road, under good satellite visibility and under poor satellite visibility. For all these quad-constellations datasets, the satellite elevation cut-off angle is set to 10°. In the implementation of the quad-constellation GNSS-SPP algorithm with mitigation of colored noise, the window size will have an effect on the position solution. In order to analyze this effect, different window sizes are adopted for testing using observations under the good satellite visibility condition. The root mean squares (RMSs) of the 3D positioning errors for different window sizes are shown in Table 1. It is obvious that the RMS error is the smallest when the window size is set to six. Therefore, the window size is empirically set to six in this study.

### 3.1. SPP Result Analysis under Different Satellite Visibility Conditions

**Figure 4:**
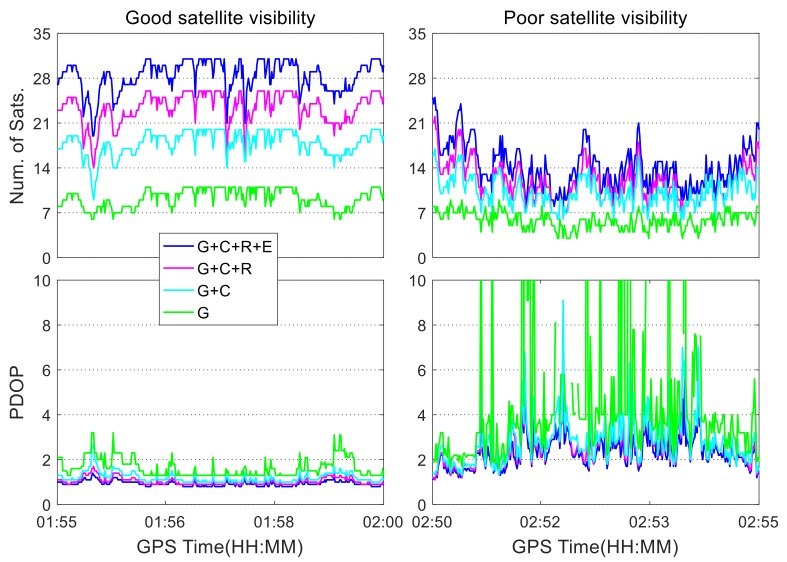
Number of satellites and position dilution of precision (PDOP) values under different satellite visibility conditions.

For the navigation test shown in Figure 2, the SPP results are presented under different satellite visibility conditions. The number of satellites and position dilution of precision (PDOP) values for different constellation combinations are shown in Figure 4, in which “G” represents a single GPS system, “G + C” is a combination of GPS and BDS, “G + C + R” is a combination of GPS, BDS, and GLONASS, and “G + C + R + E” denotes the quad-constellation combination of GPS, BDS, GLONASS, and Galileo. For the quad-constellation combined navigation on the open-sky road, the number of satellites under good satellite visibility conditions is between 25 and 30, with an average of 28.3; while their corresponding PDOP values are between 0.8 and 1.0, with a mean value of 0.9. These numbers indicate good observation conditions. In comparison, on the road with poor satellite visibility, as indicated by the red marks in Figure 2, the total number of visible satellites from all four constellations is between 10 and 20, with an average of 14.2, and their PDOP mean value is 2.4, with a maximum value of about 7.0. The increase of the PDOP value will amplify the measurement noise in the state estimation. The poor visibility road is located on the main campus of Central South University, where the GNSS signals are easily blocked by trees and buildings. As seen from the right subplot, the quad-constellation combination greatly improves the satellite visibility and decreases PDOP values when compared with the single constellation on the signal blocked road.

**Figure 5:**
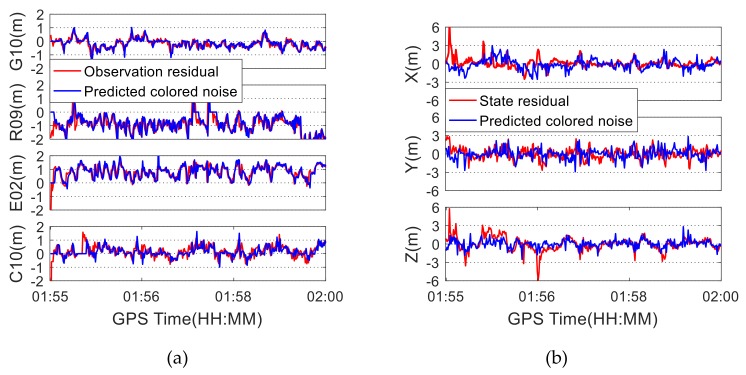
Residuals and colored noise under good satellite visibility condition: (**a**) Observation residuals and predicted colored noise; (**b**) State residuals and predicted colored noise.

Using the datasets under good satellite visibility, the colored noise model based on the approach described in Equations (6)–(12) was established and the observation colored noise and the state colored noise were obtained following the processing procedure shown in Figure 1. As a representative, the acquired observation colored noise for four satellites from four different constellations, as well as state colored noise, are shown in Figure 5. In this figure, "residual" represents the residual vector in the Kalman filtering process, while "predicted noise" represents the colored noise predicted by the fitting function model. As can be seen from Figure 5a, the observation colored noise is consistent with the original residual sequence, indicating that the function fitting model can effectively model and predict the colored noise. The root mean squares (RMSs) of the differences between the observation residuals and the predicted colored noise are 0.64 m, 0.42 m, 0.25 m, and 0.57 m for satellites C10, E02, G10, and R09, respectively. The state noise in the three coordinate components is obtained, as shown in Figure 5b. The predicted state colored noise is basically consistent with the state residual. The RMS values of the differences between the state residuals, and the predicted state colored noises are 1.19 m, 1.20 m, and 1.08 m in the X, Y, and Z directions, respectively. The state colored noise exhibits larger fluctuation than the observation colored noise due to relatively larger state residuals.

**Figure 6:**
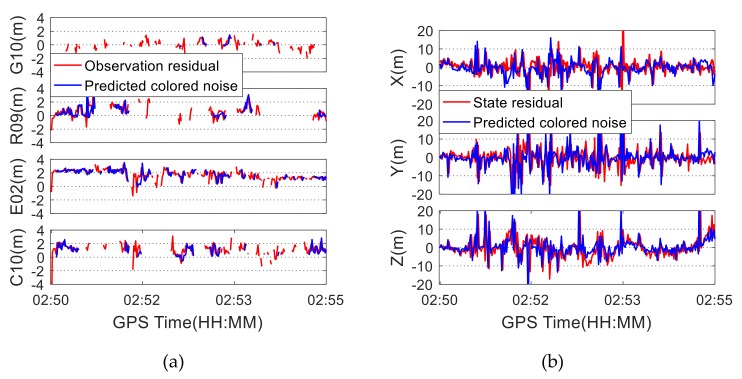
Residuals and colored noise under poor satellite visibility condition: (**a**) Observation residuals and predicted colored noise; (**b**) State residuals and predicted colored noise.

Similar to Figure 5, Figure 6 shows the residuals and the predicted colored noise using the datasets under poor satellite visibility conditions. It can be seen from Figure 6a that the observations are not complete due to the occlusion of obstacles, and so the observation residuals appear to be discontinued. However, the quad-constellation combination can make up the deficiency of observation data caused by the absence of visible satellites. As a result, the positioning results seem to be more continuous. Although the observation colored noise can be predicted, it is not as good as the predicted effect when the satellite visibility is good. The RMSs of the differences between the predicted observation colored noise and the observation residuals are 0.76 m, 0.58 m, 0.39 m, and 1.02 m for satellites C10, E02, G10, and R09, respectively. As can be seen from Figure 6b, when the visibility of the satellite is poor, the state residual is significantly larger, and the predicted effect of the colored noise is affected. This is because when the satellite visibility becomes worse, the measurement noise is magnified by the poorer satellite geometry, resulting in larger state residuals. Consequently, the predicted colored noise is affected.

**Figure 7:**
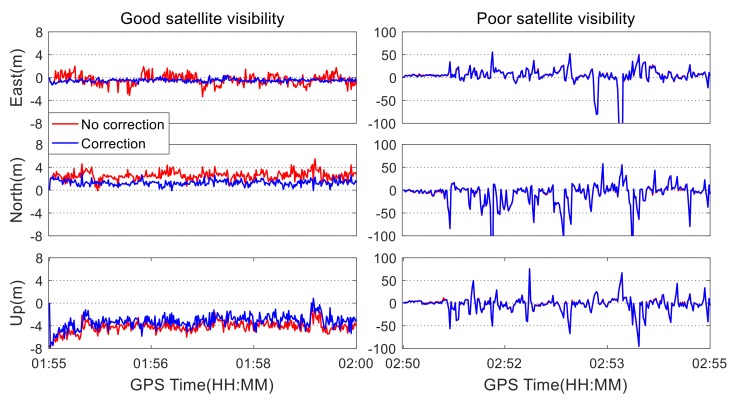
Quad-constellation positioning errors with and without the correction of colored noise under different satellite visibility conditions.

Under different satellite visibility conditions, the quad-constellation SPP solutions are obtained using the Kalman filter. The RTK solutions are used as coordinate references. The positioning errors with or without applying colored noise correction are shown in Figure 7. As seen from the figure, when the satellite visibility is good, the positioning error is close to zero after applying the colored noise correction. In comparison, the positioning errors are more fluctuant if the colored noise effect is not taken into account. As seen from Table 2, the positioning accuracy increases by 19.1%, 26.5%, and 35.3%, respectively, in three coordinate components, and the three-dimensional position accuracy increases by 27.3% after considering the effect of the colored noise. When the satellite visibility is poor, the horizontal and vertical position errors become larger. As a result, the improvement of the positioning accuracy is not obvious after applying the colored noise correction, as seen from Figure 7 and Table 2. The reason is that the satellite geometry condition plays a more important role than the colored noise in the limited satellite visibility conditions. Simultaneously, the discontinued observations affect the establishment of the functional fitting model of the colored noise. 

**Figure 8:**
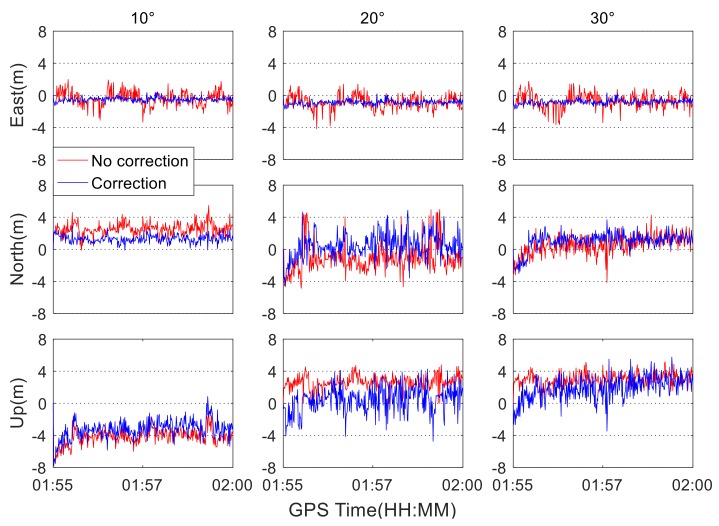
Quad-constellation positioning errors with and without the correction of colored noise at different elevation cut-off angles.

It is well known that different satellite elevation angles will affect the signal-to-noise ratio of observation data. The higher the satellite elevation angle is, the larger the signal-to-noise ratio. To test the impact of the satellite elevation mask angles on positioning solutions with correction of the colored noise, the quad-constellation datasets under the good satellite visibility conditions are processed by setting different elevation cut-off angles, and the influence of the colored noise on the quad-constellation positioning solutions is evaluated. When the elevation cut-off angles are set to 10°, 20°, and 30°, the positioning errors with and without the colored noise correction are shown in Figure 8. The corresponding RMS statistics of positioning errors are displayed in Table 3. 

As can be seen from Figure 8, the positioning accuracy increases as the elevation cut-off angle increases. This is because the residual errors, such as ionospheric and atmospheric delay errors, are smaller when satellite elevation angles are higher, which leads to an improvement of positioning accuracy. It can be seen from Table 3 that the 3D positioning accuracy improvement rate gradually decreases from 27.3% to 16.6% with the increment of the elevation cut-off angle. This is easily understood, since the magnitude of colored noise is decreased with the increment of elevation cut-off angle in the case of sufficient satellite number of quad-constellations.

### 3.2. Positioning Result Analysis Under Different Road Conditions

**Figure 9:**
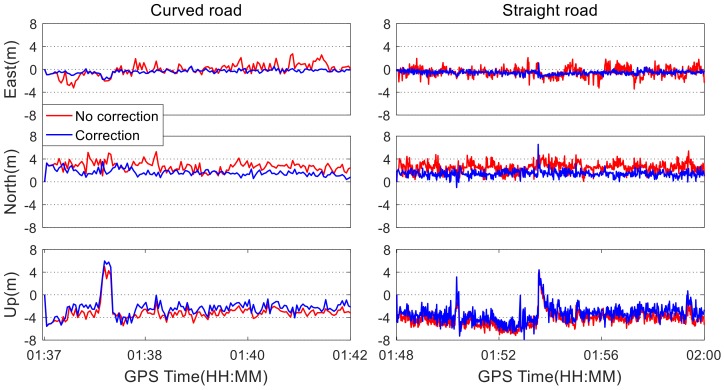
Quad-constellation SPP errors with and without correction of colored noise under different road patterns.

Kinematic data collected for different road patterns were processed to evaluate the influence of colored noise on the positioning accuracy, including the straight and curved road patterns. These different road patterns are shown in Figure 2. Since the used standard Kalman filter is a linear model, the estimated position vector at the current epoch is a sum of the position solution at the last epoch and the change value at the current epoch. Therefore, it is generally considered that the obtained accuracy on the straight road is higher than that on the curved road. Using the colored noise mitigation algorithm developed in Section 2, the positioning errors of the quad-constellation combined SPP with and without colored noise correction are obtained under different road patterns, respectively. They are shown in Figure 9.

It can be seen from Figure 9 that positioning errors can be effectively reduced after correcting the colored noise for both the curved and straight road patterns. On both the straight and the curved roads, some obviously larger positioning errors occurred, especially in the vertical coordinate component. This is because the bumped road surface degrades the state prediction accuracy in the Kalman filter. Simultaneously, at the moments that these large positioning errors appear, the observation residuals and state residuals are abnormally larger, leading to an inaccurate modeling of the colored noise. As a result, the correction of the colored noise cannot improve the positioning performance. Table 4 shows the RMS statistics of the quad-constellation SPP errors with and without correction of colored noise under different road patterns. The positioning accuracy on the straight road after correcting the colored noise is improved by 16.8%, 23.1%, and 35.2% in the east, north, and up directions, respectively. The three-dimensional position accuracy is improved by 22.1%. On the curved road, the positioning accuracy after taking the colored noise into account is correspondingly improved by 16.3%, 25.7%, and 44.5% in the three directions, respectively. The three-dimensional positional accuracy is improved by 25.7%. The horizontal position accuracy is comparable for both straight and curved road conditions, suggesting that the state equation has well-modeled the car kinematic under the two different road patterns. Further, the improvement rates of the positioning accuracy after correcting the colored noises are also comparable with both straight and curved roads.

## 4. Conclusions

In the navigation, most observation noise and state noise belongs to colored noise, due to the complex observation environment and unpredictable dynamics. The colored noise can significantly affect the positioning accuracy of the navigation solutions. Based on observation residuals and state prediction residuals, this paper develops a colored noise model to mitigate the colored noise in the quad-constellation SPP. Kinematic positioning experiments under different satellite visibility conditions and road patterns were conducted to test the influence of the colored noise on the positioning accuracy of quad-constellation navigation. The experimental results show that the colored noise model can effectively predict the colored noise and then mitigate its effect on the positioning accuracy. The three-dimensional positioning accuracy can be improved by 27.3% under the good satellite visibility condition. When satellite visibility is poor, the large residual errors have a side effect on the acquisition of the colored noise. As a result, the improvement of the positioning accuracy after correcting the colored noise is not significant. For different satellite elevation cut-off angles, the colored noise contained in the observations decreases with the increase of elevation angles. Consequently, the improvement of positioning accuracy after considering colored noise gradually decreases from 27.3% to 16.6% with the increment of the elevation cut-off angle. In the case of different road patterns, the positioning accuracy after considering colored noise is improved by over 22% on both straight and curved roads.

## Figures and Tables

**Table 1:** RMS statistics of positioning errors with different window sizes (m).

**Window Sizes**	4	5	6	8	10	12
**3D**	4.33	4.31	4.24	4.32	4.36	4.37

**Table 2:** Root mean square (RMS) statistics of positioning errors with and without colored noise correction under different satellite visibility conditions (m).

Satellite Visibility	Colored Noise	East	North	Up	3D	Improvement Rate
Good	No correction	0.96	2.65	4.31	5.15	27.3%
	Correction	0.57	1.37	3.43	3.74	
Poor	No correction	27.17	30.17	18.22	44.50	0.4%
	Correction	27.11	30.05	18.06	44.32	

**Table 3:** RMS statistics of quad-constellation positioning errors with and without the correction of colored noise at different elevation cut-off angles (m).

Elevation Cut−Off Angle	Colored Noise	East	North	Up	3D	Improvement Rate
10°	No correction	0.96	2.65	4.31	5.15	27.3%
	Correction	0.57	1.37	3.43	3.74	
20°	No correction	1.21	2.08	2.72	3.64	21.1%
	Correction	0.95	1.61	2.17	2.87	
30°	No correction	1.15	1.31	3.08	3.54	16.6%
	Correction	0.89	1.50	2.38	2.95	

**Table 4:** RMS statistics of quad-constellation SPP errors with and without correction of colored noise under different road patterns (m).

Road Patterns	Colored Noise	East	North	Up	3D	Improvement Rate
Curve	No correction	1.04	2.83	3.43	4.57	25.7%
	Correction	0.52	1.76	2.85	3.39	
Straight	No correction	0.91	2.70	4.50	5.32	22.1%
	Correction	0.65	1.53	3.72	4.08	
